# UCSC Genome Browser enters 20th year

**DOI:** 10.1093/nar/gkz1012

**Published:** 2019-11-06

**Authors:** Christopher M Lee, Galt P Barber, Jonathan Casper, Hiram Clawson, Mark Diekhans, Jairo Navarro Gonzalez, Angie S Hinrichs, Brian T Lee, Luis R Nassar, Conner C Powell, Brian J Raney, Kate R Rosenbloom, Daniel Schmelter, Matthew L Speir, Ann S Zweig, David Haussler, Maximilian Haeussler, Robert M Kuhn, W James Kent

**Affiliations:** 1 Genomics Institute, University of California Santa Cruz, Santa Cruz, CA 95064, USA; 2 Howard Hughes Medical Institute, University of California Santa Cruz, Santa Cruz, CA 95064, USA

## Abstract

The University of California Santa Cruz Genome Browser website (https://genome.ucsc.edu) enters its 20th year of providing high-quality genomics data visualization and genome annotations to the research community. In the past year, we have added a new option to our web BLAT tool that allows search against all genomes, a single-cell expression viewer (https://cells.ucsc.edu), a ‘lollipop’ plot display mode for high-density variation data, a RESTful API for data extraction and a custom-track backup feature. New datasets include Tabula Muris single-cell expression data, GeneHancer regulatory annotations, The Cancer Genome Atlas Pan-Cancer variants, Genome Reference Consortium Patch sequences, new ENCODE transcription factor binding site peaks and clusters, the Database of Genomic Variants Gold Standard Variants, Genomenon Mastermind variants and three new multi-species alignment tracks.

## INTRODUCTION

The University of California Santa Cruz (UCSC) Genome Browser (1) is a web-based viewer for genome sequence data and annotations. The UCSC Genome Browser team has steadily added data and software features to the website since first coming online in July 2000, and currently hosts 206 assemblies from 105 species. We continue to add new species and assemblies, but, as most of our user base focuses on human and mouse, the vast majority of annotation data are focused on those two species. In addition to displaying and interacting with data hosted on UCSC servers, users can also upload and display their own data in the form of custom tracks (a single set of annotations) or track hubs (externally hosted collections of tracks). We encourage track hub creators to contact us about having their hubs added to our list of public track hubs.

A large part of our recent effort has been in making track hubs easier to use and configure, as well as encouraging researchers to submit their track hubs to the list of ‘public hubs’ (accessible from the blue top navigation bar as ‘My Data → Track Hubs’), so that other researchers can more easily discover and access their data. A track hub consists of data files and configuration files that organize the data into annotation tracks, adding labels and descriptions and optionally grouping related datasets into composite tracks. A guide to the various options available for creating a track hub is available in the Track Hub Database Definition page (https://genome.ucsc.edu/goldenPath/help/trackDb/trackDbHub.html).

Along with displaying and hosting data, our site also provides a number of tools for interacting with, comparing and downloading those data. Some, such as the Table Browser (2) and BLAT (3), are available for direct use on the web. Other tools are available for download—we provide a directory of over 200 utilities (‘Downloads → Utilities’) that can be used to download and examine data files, or convert between formats. We also maintain a public MySQL server (‘Downloads → MySQL Access’) for accessing our annotation data, and with this update have added a RESTful API (‘Downloads → REST API’), both of which can be queried to download and access the data we host. The API is described in more detail below.

We continue to support local ‘mirroring’ of the Genome Browser software and data (‘Mirrors → Mirroring Instructions’). The Genome Browser in a Box (GBiB) (4) is a fully pre-configured virtual machine that is easy to set up and run. For enterprise solutions, the Genome Browser in the Cloud (GBiC) is a tool that automates the setup of a mirror instance on a bare-metal or cloud server. Both provide dynamic access to the data on UCSC’s servers as well as the option of downloading data locally for offline use. This last option may be useful for navigating clinical or proprietary data or using the Browser behind a restrictive firewall.

## ANNOTATIONS AND VISUALIZATIONS

Over the past year, we have continued to update and add tracks and assemblies. A full list of the new data is available in Supplementary Table S1 (assemblies) and Supplementary Table S2 (tracks), but a few items deserve special mention due to their accompanying visualization features: the TCGA Pan-Cancer dataset and the accompanying new ‘lollipop’ display mode, the GeneHancer database of regulatory elements and the accompanying improvements to the recently introduced interact format, and the Tabula Muris single-cell RNA expression dataset and accompanying single-cell browser. We have also expanded our support of human genome assembly patches released by the Genome Reference Consortium, and have added annotations from many tracks onto these new sequences to better support researchers studying human variation.

### GeneHancer and interact track format improvements

**Figure 1.**
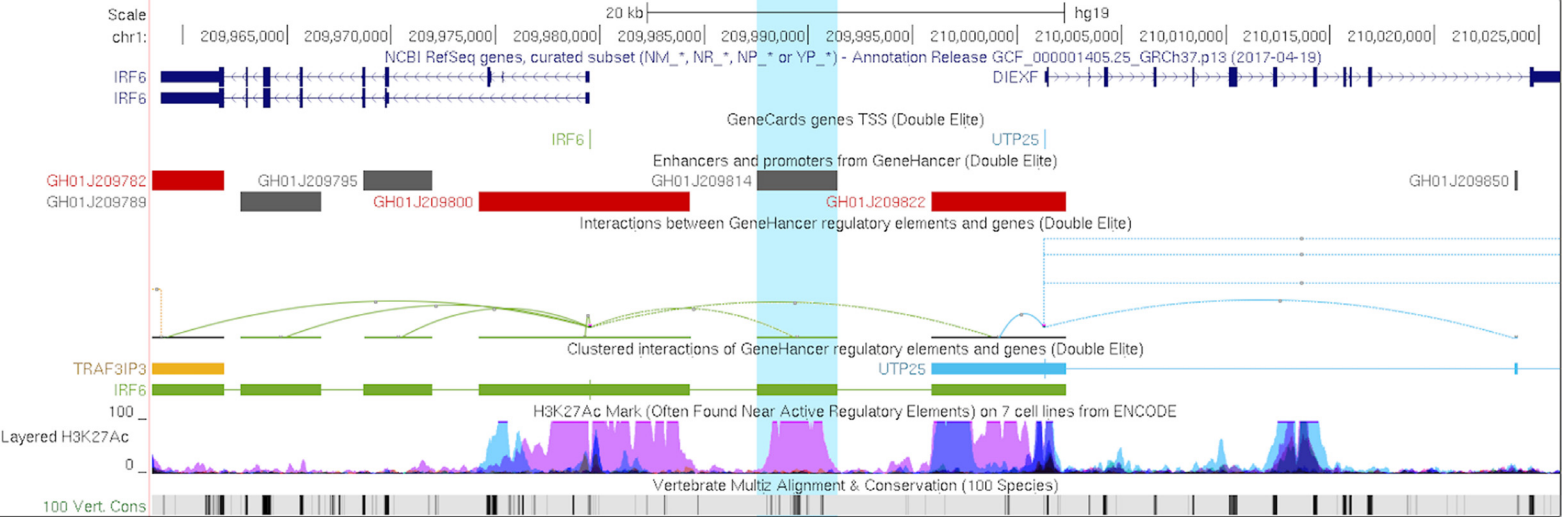
The GeneHancer track set and the interaction track format with hills instead of the default valleys. The GeneHancer track relates enhancer and promoters to their interactions with nearby genes. In this display, a highlighted GH01J209814 enhancer is associated with the gene IRF6 (interferon regulatory factor 6) located about 10 kb upstream.

Last year (5) we introduced a new ‘interact’ track format that draws connecting arcs between pairs of genomic regions. This year brings the release of the first native track utilizing this format, the GeneHancer track from the GeneCards group (6). GeneHancer is a database of human regulatory elements (enhancers and promoters) and their inferred target genes. These elements were synthesized from over 1 million regulatory elements obtained from seven genome-wide databases: ENCODE (7), Ensembl (8), FANTOM5 (9), VISTA (10), dbSUPER (11), EPDNew (12) and UCNEbase (13). The highly filtered ‘double elite’ dataset is displayed by default, and the full dataset can be enabled from the track configuration page. This track is available on the GRCh37/hg19 and GRCh38/hg38 assemblies. In support of this track, a number of enhancements were made to the interact track format, including an inverted view (hills instead of valleys, Figure 1), ‘pack’ and ‘squish’ visibility modes, and a cluster mode, which combines directional interactions with the same target or source into a single item.

### TCGA Pan-Cancer data and the new ‘lollipop’ plot display

**Figure 2.**
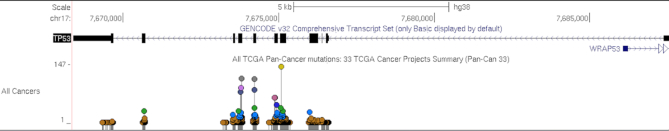
The TCGA Pan-Cancer track at the TP53 locus. The lollipop display showcases SNPs found in cancers that were not present in normal tissue. Lollipops are shaded according to their score relative to the other SNPs in the current view. The tall yellow and gray lollipops stand out to reveal hotspots indicated across ∼120 people and multiple cancers.

The new TCGA Pan-Cancer mutations track for the hg38 assembly presents somatic variants called from 33 whole-genome sequencing projects, as part of The Cancer Genome Atlas. Variant calls were downloaded from the Genomic Data Commons TCGA portal (14) and transformed into one track per cancer type as well as one merged track. As shown in Figure 2, this track features a new ‘lollipop’ plot display type, where the position of a lollipop circle corresponds to the genomic coordinate of the variant, and the height of the lollipop stem reflects the number of samples in which the variant was found. Lollipop circles are shaded according to their score relative to the other variants in the current viewing window. This mode allows variants common to many samples (or high-scoring in the general case) to stand out in a sea of potentially spurious cancer mutations. The TCGA Pan-Cancer track also supports filtering of items by sample count, gender and TCGA Project ID.

### Tabula Muris and the single-cell browser

The GRCm38/mm10 assembly features a new native track, Tabula Muris (15). The Tabula Muris transcriptome compendium contains single-cell sequencing data from roughly 100 000 cells and 20 different organs/tissues. The data have been organized into three separate tracks: an overview bar graph track of median expression per cell type, an individual wiggle graph of the number of reads at a particular base pair, and a track showing an analysis of splice junctions.

**Figure 3.**
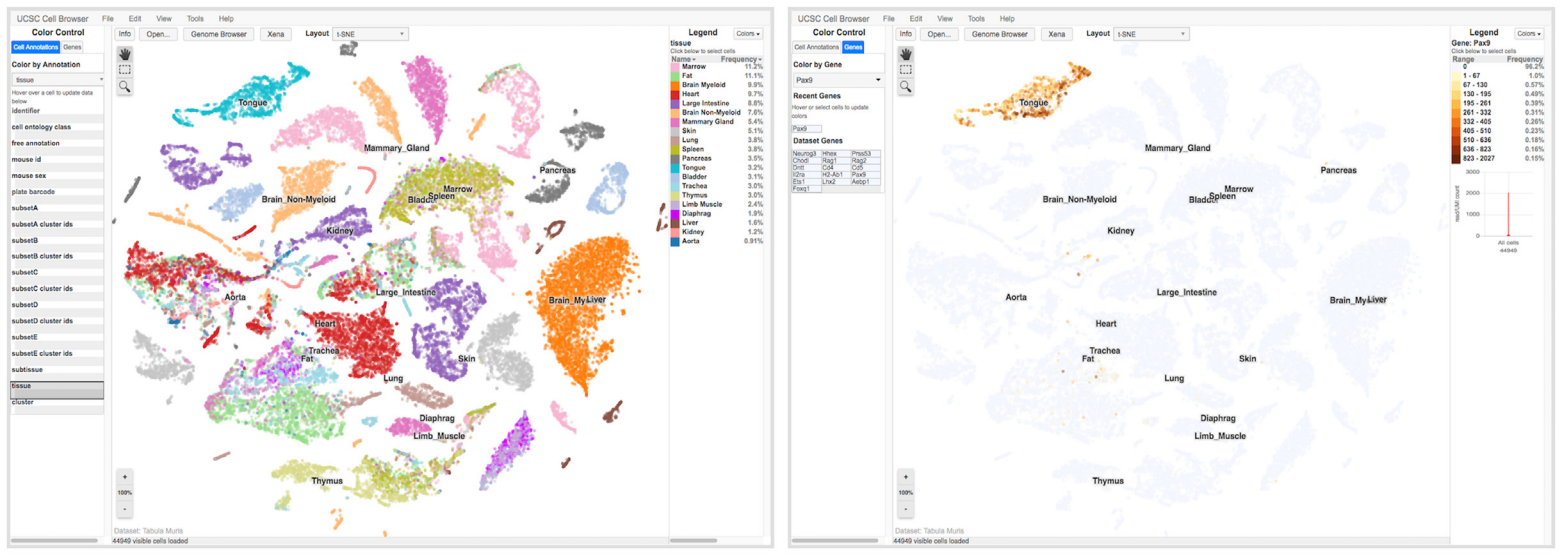
The single-cell browser. The left-hand panel shows the default view upon loading the Tabula Muris dataset in the cell browser. Cells are shaded according to which tissue cluster they belong to, and each tissue cluster is labeled accordingly. Hovering the mouse pointer over an individual cell populates the fields on the left-hand panel with the metadata associated with each cell. The right-hand panel shows the result of filtering all of the cells for Pax9 gene expression.

Along with this dataset, we have created a new tool for visualization of single-cell data, termed the ‘single-cell browser’ (Figure 3). The single-cell browser is available from the landing page through the menu (‘Projects → UCSC Cell Browser’), or directly via https://cells.ucsc.edu. The single-cell browser presents an interactive display from the data produced by a standard Cellranger, Seurat (16) or SCANPY (17) run (Figure 3, left panel). This tool supports clicking or hovering on cells to obtain metadata about each cell, coloring cells by gene (Figure 3, right panel), and clicking to show cluster-specific marker genes. In addition to the Tabula Muris data, there are 16 other single-cell experiments available to explore on the single-cell browser website.

### Human patch sequences

With the Genome Reference Consortium (GRC) deciding to indefinitely postpone the next coordinate-changing human assembly GRCh39 (https://www.ncbi.nlm.nih.gov/grc/human), we have committed to modifying how we handle the patch sequences released by the GRC. Previously, when a new patch sequence release was announced, tracks were created against the target assembly (e.g. hg38) that allowed these sequences to be viewed in their genomic context. Now, for the hg38 assembly only, we are incorporating the patch sequences directly into our assembly database with remapped or recomputed annotations. This has numerous consequences: BLAT results now include matches on _alt and _fix sequences, position searches may lead to new sequences in addition to initial assembly release sequences, and some tracks include annotations on new sequences. In the case of certain third-party datasets (like GENCODE or RefSeq), we now directly incorporate their existing patch sequence annotations. Annotations computed by us, such as RepeatMasker, CpG Islands, and Augustus, have been expanded to include the patch sequences. We have also remapped annotations from main chromosomes to patch sequences for several popular tracks such as GTEx Genes and ENCODE Regulation. Tracks that include annotations on patch sequences in GRCh38.p12 are marked with a ‘P12’ icon next to the track label in the track controls section. To avoid breaking pre-existing pipelines, the sequence files available in the hg38 bigZips download directory (e.g. https://hgdownload.soe.ucsc.edu/goldenPath/hg38/bigZips/hg38.fa.gz) will remain unchanged. Instead, new subdirectories have been added in bigZips: ‘p12’ for files that include patch sequences from the GRCh38.p12 release, ‘initial’ to explicitly provide unchanged files, and ‘latest’ to provide stable links to files from the most recent patch release to support pipelines that do include patches. In addition to the extended data, the multiregion feature (‘View → Multi-Region’) now supports autocomplete search for fix and patch sequences to facilitate the viewing of alternate sequences in their genomic context. More information about our incorporation of sequence patches can be found at http://genome.ucsc.edu/blog/patches/.

### ENCODE transcription factor binding site peaks and clusters

The ENCODE Transcription Factor Binding Site Peaks and Clusters track sets show regions of transcription factor binding derived from a large collection of ChIP-seq experiments performed by the ENCODE project between February 2011 and November 2018 (ENCODE phases 2 and 3). The tracks are organized into two types: the Peaks set contains the underlying ChIP-seq peaks and can be optionally filtered by cell type or transcription factor, while the Clusters track provides a summary display of occupancy regions for each cluster. This track is available for hg38 and hg19.

### DGV Gold Standard Variants

The Database of Genomic Variants (18) is a curated set of large structural variants that appear in healthy individuals. The Gold Standard set is created by starting with all of the variants in DGV that are found in at least two different studies and at least two different samples, filtering out low-quality variants and then merging what is left according to a 50% minimum overlap. The variants are then combined into a ‘boxplot’-like record, where the highest quality variant in the cluster forms the ‘inner box’, and the whiskers define the maximal coordinates of the copy number variants. This track is available for hg19.

### Genomenon Mastermind

The Mastermind search engine from Genomenon (https://www.genomenon.com/mastermind) mines full-text publications for disease–gene–variant associations. We have created a track on hg19 that shows variants that have been indexed by the search engine, with links to Genomenon for more details such as references in which the variant was reported and associated with disease.

### Gene set updates

This year we have continued to add Ensembl gene sets and GENCODE track sets as they became available, most recently adding Ensembl v95 for most assemblies, GENCODE v31 for hg19 and hg38, and M22 for mm10. Along with updating the GENCODE track sets, we have also updated the default gene set for hg38 to GENCODE v29, and switched to using the ENST* transcripts as the default transcript names instead of the previous uc* identifiers. Finally, there are two new tracks in the NCBI RefSeq track set for the hg38 and hg19 assemblies: a ‘Diffs’ track that shows differences between the transcript sequences provided by RefSeq and the reference genome, and a track for transcripts that are selected as the most clinically relevant by the Human Gene Mutation Database (HGMD) curators (19). For more information about the different gene sets available, see the following page: https://genome.ucsc.edu/FAQ/FAQgenes.html.

### Multiple alignments for chicken, *Drosophila* and *Caenorhabditis elegans*

This year we have created three new multiple alignment tracks: one for chicken (GRCg6a/galGal6) that features 76 other vertebrates, one for *Drosophila melanogaster* (BGDP Release 6/dm6) that features 123 other insects and one for *C. elegans* (WBcel235/ce11) that features 134 other worms.

### New public track hubs

A number of groups have created new hubs to share with the general UCSC Genome Browser user community, notably UniBind (20), DASHR v2.0 (21), human p53 Binding and Expression Resource (22), Primate x4 NeuroDiff and Human CRISPRa (23) and the Vertebrate Genomes Project assemblies (24).

## SOFTWARE AND TOOL IMPROVEMENTS

### BLAT against all genomes

**Figure 4.**
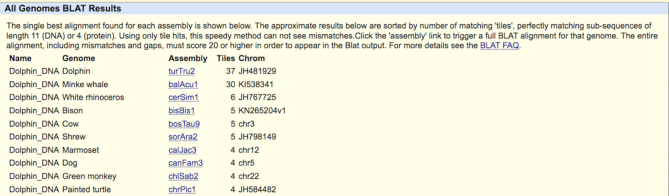
The BLAT All search results for a chunk of dolphin DNA show that the majority of potential hits apply only to the source turTru2 (*Tursiops truncatus*) dolphin assembly, but also suggest interesting matches to minke whale.

The BLAST-like Alignment Tool (BLAT) was one of the first features of the Browser, and remains one of the most popular tools on the site. Normally used to discover the exon–intron structure of a piece of mRNA or to find sequence similarity across a particular genome sequence, BLAT (‘Tools → Blat’) can now, with a single click, search across all of the default organisms available in the UCSC database as well as any attached assembly hubs. Each of over 100 genomes is searched in parallel, resulting in a quick return of matched ‘tiles’, sorted by the organism with the most matches (Figure 4), as a heuristic indicator of which species are more likely to have true alignments. Clicking into each organism causes a full BLAT search to execute for that organism and leads to the standard BLAT results section.

Instead of carrying out a full BLAT search against each genome, the BLAT All feature only checks for matching 11-mers (or 4-mers in the case of protein queries), which are called ‘tiles’. Mismatches and gaps are not accounted for, which substantially speeds up the search while still allowing users to get an overall idea of sequence homology. Results can then be explored further with the standard BLAT interface. For more information on the BLAT All feature, we have updated the BLAT documentation available here: https://genome.ucsc.edu/FAQ/FAQblat.html#blat9.

### REST API

Previously, users who wanted to download track or sequence data would need to grab the dataset in its entirety from our downloads server, or use the Table Browser's graphical interface. Unfortunately, scripting against these resources was never practical for dynamic queries like getting track data for >1000 regions or accessing UCSC data via R/Python/Perl or another programming environment. To better support those use cases, we have created a RESTful API that returns JSON-formatted data for a variety of data retrieval queries. The API is accessed via the URL https://api.genome.ucsc.edu and has two primary functions: listing available datasets and downloading data from said datasets. Notable endpoints include /list/tracks/ for listing all of the tracks for a given database or hub, and /getData/sequence/ and /getData/track/ for obtaining genome sequence and annotation data, respectively. For a user guide including the full list of endpoints, please see the documentation at https://genome.ucsc.edu/goldenPath/help/api.html.

### Session Custom-Track backups

We have also made improvements to the Sessions (‘My Data → My Sessions’) tool. Primarily, users can now download all of the custom tracks currently loaded in their browsing session by visiting the Sessions page and using the ‘Save Custom Tracks’ button in the ‘Save Settings’ section. The custom track data will all be combined into one archive file for download, and then, if needed, immediately reloaded back into the Genome Browser. We hope this will be convenient for users who experiment with large numbers of custom tracks before finally deciding on a session to share for a publication or send to a colleague. For more information on this backup feature, see the ‘Creating a session’ section of the Sessions help page (https://genome.ucsc.edu/goldenPath/help/hgSessionHelp.html). In addition to the backup feature, sessions can now be loaded via significantly shorter links that are more informative for publications and for emailing to colleagues. The new URL format is https://genome.ucsc.edu/s/username/sessionname.

## OUTREACH AND CONTACT INFORMATION

In the last year, the Genome Browser's training team provided 30 seminars and workshops to help users take advantage of the latest features. We have now presented trainings in 27 countries and 34 states and Canadian provinces (http://bit.ly/ucscMap). Outreach is supported by a blog (http://genome.ucsc.edu/blog) and by updates to the training documentation (https://genome.ucsc.edu/training/) with links to videos and in-depth descriptions of new Browser features. The training page also includes information on how to submit a request for a workshop and where future workshops may be scheduled.

General contact information for the UCSC Genome Browser can be found at https://genome.ucsc.edu/contacts.html, including information on accessing our email support list and an archive of previously answered mailing list questions. UCSC also maintains mirrors in Germany and Japan with the gracious assistance of Bielefeld University, Germany, and RIKEN, Japan. Those sites can be found at https://genome-euro.ucsc.edu and https://genome-asia.ucsc.edu.

## FUTURE PLANS

The UCSC Genome Browser team has several major goals for the coming year. We plan to continue development of the single-cell browser and to add more support for single-cell sequencing data. We also plan to develop two new visualizations, a heatmap display for Hi-C chromatin interactions and a phased trio display for personal genomics data. On the data side, we plan to add annotations onto the mm10 and hg19 patch sequences and to support visualization of data from the NCBI Sequence Read Archive.

## Supplementary Material

gkz1012_Supplemental_File
