# Supplementary material for: UCSC Genome Browser enters 20th year
Source: Nucleic Acids Res. 2019 Nov 6;48(D1):D756–61. doi: 10.1093/nar/gkz1012 (PMC7145642; doi:10.1093/nar/gkz1012)
Supplement: gkz1012_Supplemental_File [file gkz1012_supplemental_file.pdf]

Supplementary Table 1. Assemblies added in the last year

Organism	NCBI Accession	Provider	UCSC Name
Cow	GCF_002263795.1	USDA ARS	bosTau9
Horse	GCF_002863925.1	University of Louisville	equCab3
Baboon	GCF_000264685.3	Human Genome Sequencing Center	papAnu4
Chinese Hamster Ovary	GCA_900186095.1	Eagle Genomics Ltd	criGriChoV2
Cat	GCF_000181335.3	Genome Sequencing Center (GSC) at Washington University (WashU) School of Medicine	felCat9
Chicken	GCF_000002315.5	Genome Reference Consortium	galGal6
Orangutan	GCF_002880775.1	University of Washington	ponAbe3
Chimp	GCF_002880755.1	University of Washington	panTro6
Sheep	GCF_000298735.2	International Sheep Genome Consortium	oviAri4
A. gambiae	GCF_000005575.2	The International Consortium for the Sequencing of Anopheles Genome	anoGam3
Garter Snake	GCA_001077635.2	The Genome Institute at Washington University School of Medicine (WUGSC)	thaSir1

Supplementary Table 2. Annotation tracks added or updated within the last year.

KEY: N = new, NU = new & updated, U = updated, AU = automatically updated

**Track Name**	**New/Update Status**	**Human assemblies**	**Mouse assemblies**	**Other assemblies**
ENCODE TF Clusters	N	hg38, hg19		
ENCODE TF ChIP	N	hg38, hg19		
GeneHancer	N	hg38, hg19		
RefSeq HGMD	N	hg38, hg19		
dbSNP 151	N	hg38, hg19		
HGMD Variants	U	hg38, hg19		
GDC Cancer	N	hg38		
GENCODE Genes v31, v29,v25	N	hg38		
MANE Transcripts v0.6	NU	hg38		
sno/miRNA	NU	hg38		
miRNA Tissue Atlas	N	hg38		
RefSeq Diffs	N	hg38		
CRISPR All	N	hg19		
Mastermind Variants	N	hg19		
DGV Gold Standard Variants	N	hg19		
Tabula Muris	N		mm10	
GENCODE Genes (VM22, VM20, VM18)	N		mm10	
CRISPR 10K	N	hg38		galGal6, bosTau9, anoGam3
77-species Conservation	N			galGal6
123-species Conservation	N			dm6
135-species	N			ce11

Conservation				
Chains & Nets (for many assemblies)	N	various	various	various
Ensembl Genes v95	N	various	various	various
Transmap v5	N	various	various	various
GenBank Updates (RefSeq Genes, ESTs, RNAs)	AU	Most assemblies updated	Most assemblies updated	Most assemblies updated
GRC Incident	AU	hg38, hg19	mm10, mm9	danRer10, danRer7, galGal5
ClinVar Nucleotide Variants	AU	hg38,hg19		
Gene Reviews	AU	hg38, hg19, hg18		
NHGRI Catalog of Published GWAS	AU	hg38, hg19, hg18		
OMIM Genes & Phenotypes	AU	hg38, hg19, hg18		
ClinGen Research	AU	hg38, hg19		
ClinVar Variants	AU	hg38, hg19		
DECIPHER	AU	hg19		
LOVD	AU	hg19		
